# The role of dedicated biocontainment patient care units in preparing for COVID-19 and other infectious disease outbreaks

**DOI:** 10.1017/ice.2020.451

**Published:** 2020-09-04

**Authors:** Jade B. Flinn, Noreen A. Hynes, Lauren M. Sauer, Lisa L. Maragakis, Brian T. Garibaldi

**Affiliations:** 1 Department of Nursing, Johns Hopkins Hospital, Baltimore, Maryland; 2 Division of Infectious Diseases, Johns Hopkins University School of Medicine, Baltimore, Maryland; 3 Department of Emergency Medicine, Johns Hopkins University School of Medicine, Baltimore, Maryland; 4 Division of Pulmonary and Critical Care Medicine, Johns Hopkins University School of Medicine, Baltimore, Maryland

## Abstract

In response to the Ebola outbreak of 2014–2016, the US Office of the Assistant Secretary for Preparedness and Response (ASPR) established 10 regional treatment centers, called biocontainment units (BCUs), to prepare and provide care for patients infected with high-consequence pathogens. Many of these BCUs were among the first units to activate for coronavirus disease 2019 (COVID-19) patient care. The activities of the Johns Hopkins BCU helped prepare the Johns Hopkins Health System for COVID-19 in the 3 domains of containment care: (1) preparedness planning, education and training, (2) patient care and unit operations, and (3) research and innovation. Here, we describe the role of the JH BCU in the Hopkins COVID-19 response to illustrate the value of BCUs in the current pandemic and their potential role in preparing healthcare facilities and health systems for future infectious disease threats.

In response to the 2014 Ebola outbreak in West Africa, the Office of the Assistant Secretary for Preparedness and Response (ASPR) created a regional network of 10 high-level isolation units (HLIUs), also known as biocontainment units (BCUs), to enhance readiness in the United States to respond to high-consequence pathogens.^1^ Although most of these units were initially formed to care for patients with suspected or confirmed infection with communicable viral hemorrhagic fever viruses, they were also designed to care for patients with other high-consequence pathogens such as Middle East respiratory syndrome (MERS), severe acute respiratory syndrome (SARS), and extensively drug-resistant tuberculosis (XDR TB).^2,3^ Notably, these US HLIUs and their clinical teams were among the first in the nation to provide care for patients infected with the SARS coronavirus-2 (SARS-CoV-2), the etiologic agent of coronavirus disease 2019 (COVID-19).^4,5^

The Johns Hopkins Biocontainment Unit (JH BCU) is the US Department of Health and Human Services (HHS) Region 3-designated HLIU treatment center, serving Delaware, Maryland, Pennsylvania, Virginia, Washington, DC, and West Virginia.^6^ The JH BCU team is a multidisciplinary and highly trained group of clinicians, frontline healthcare providers, researchers, infection prevention specialists, and emergency management personnel who have been working together since 2014 to enhance preparedness to respond and provide care for persons infected with high-consequence pathogens with high transmissibility and high mortality in the setting of few, if any, Food and Drug Administration (FDA)–approved medical countermeasures. The JH BCU has a tripartite mission that reflects the major pillars of high-consequence pathogen preparedness and response: (1) preparedness planning, education and training, (2) patient care and clinical operations, and (3) research and innovation. In its response to the COVID-19 pandemic, the JH BCU participated in readiness activities leading up to the admission of the first patient with COVID-19. The unit provided care for the first persons under investigation (PUI) and the first confirmed COVID-19 cases. They helped to establish and educate additional units and teams to safely provide care for patients suspected or confirmed to have COVID-19, and the unit currently leads clinical and translational COVID-19 research studies. The role of the JH BCU in the COVID-19 response highlights the importance of regional treatment centers in local, regional, and national efforts to combat novel infectious diseases and provides a roadmap for increasing US capacity to respond to future infectious disease outbreaks.

## Impact of the JH BCU on health system infectious disease preparedness

### Preparedness planning, education, and training prior to COVID-19

Preparedness and training are critical to the success of a high-level isolation unit. Staff must be familiar with infection prevention practices, personal protective equipment (PPE), and clinical care protocols for high-consequence pathogens, and they must maintain continuous readiness.^7^ Since 2014, the JH BCU has employed a full-time nurse educator to coordinate the training and competency assessment of >150 rostered staff members from multidisciplinary backgrounds. BCU team members represent >15 functional units in the hospital and include nurses, physicians, physician assistants, nurse practitioners, laboratory technicians, respiratory therapists, radiology technicians, and infection preventionists. The hospital infection prevention team is integral to BCU preparedness planning, protocol development, training, and assessments to ensure that evidence-based practices for infection prevention are incorporated and optimized for all BCU activities. Quarterly training and assessment sessions for all staff include updates on high-consequence pathogens, PPE donning and doffing practice, and skills training for critical protocols such as spill cleanup, PPE breaches, phlebotomy and lab specimen management, waste handling, and clinical procedures.

In addition to these unit-based activities, the JH BCU conducts quarterly training exercises in coordination with the JH Office of Emergency Management (OEM) and the Maryland State Department of Health. In the 5 years prior to COVID-19, the JH BCU successfully completed 22 preparedness exercises including 12 full functional drills, 6 table-top exercises, and 4 no-notice drills. Lessons learned from these exercises informed operational, logistic, and clinical aspects of BCU activation and helped to refine the state and regional response plans for highly infectious diseases.^8^ The BCU also activated 3 times for PUIs suspected to have viral hemorrhagic fever, which further informed care protocols.

In the 18 months prior to its activation for COVID-19, the JH BCU designed and implemented a pilot education program supported by the Maryland Department of Health that aimed to address Ebola and other emerging infectious disease preparedness gaps for frontline and special pathogen assessment hospitals throughout the state. The primary goal of the program was to improve Maryland frontline healthcare worker knowledge and skills for the identification and isolation of PUIs suspected to be infected with high-consequence pathogens. The program also sought to foster relationships between healthcare facilities and state public health agencies for effective notification and care transitions of patients infected with high-consequence pathogens.

During the pilot program, the BCU team delivered in-person training sessions at 16 hospitals for a diverse audience of >160 clinicians, local public health representatives, emergency management personnel, and hospital staff. These sessions covered the basics of infection control and prevention and emphasized the “identify, isolate, and inform” strategy for responding to infectious diseases.^9^

In addition to its role in local and regional preparedness, the BCU collaborates within a national network of treatment centers coordinated by the National Emerging Special Pathogen Training and Education Center (NETEC). This network addresses training and preparedness beyond the state and region, shares best practices for high-level infectious disease containment, and contributes to the growing science of containment care.^10^ The JH BCU has cohosted regional symposia and training events with NETEC with a particular focus on protocol development and practical, hands-on training of frontline staff and administrators.

These education and training activities prepared the BCU team to care for the first patients with COVID-19 at JHH. They also led to improved infection control practices across the health system as BCU team members shared their training and expertise with their home units and provided infection prevention and preparedness training to colleagues across the hospital and health system.^11^

### Preparedness, education, and training during the COVID-19 response

As the COVID-19 pandemic emerged at the end of 2019, the JH BCU team pivoted frontline training sessions to specifically address the need for area hospitals to operationalize surge capacity, implement robust screening for respiratory symptoms and recent travel history, and develop PPE airborne and droplet precaution ensembles and use plans. The program culminated in a statewide symposium on high-consequences pathogens on February 20, 2020, with representation from every county in the state of Maryland, just 9 days before the BCU activated for its first COVID-19 PUI. This adaptive educational experience established the BCU team as a valuable preparedness and training asset that could design and deliver expedited high-consequence pathogen training focused on infection prevention and PPE best practices.

Even as the JH BCU team actively cared for the first COVID-19 PUIs and patients confirmed to have COVID-19 at JH Health System in February–March 2020, the team helped to plan a sustainable and safe model for healthcare workers to care for PUIs and patients with COVID-19 in other non-BCU clinical units. The air handling systems on these clinical units were modified by the facilities department to have the negative air pressure, filtration, and ventilation needed to safely provide care for patients with COVID-19. These units were termed “biomode” units. In collaboration with healthcare epidemiology and infection control (HEIC) staff, the BCU team developed and implemented a just-in-time PPE training program which blended video content with hands-on skills sessions led by BCU staff. Over 4 days in March 2020, the BCU and HEIC teams trained 150 healthcare workers including clinical educators, clinical support staff, providers, and nurses in proper PPE donning and doffing techniques. These staff became ambassadors and a resource to reinforce PPE best practices throughout the hospital. The role of safety officer, a team member who assists with the safe donning and doffing of personnel working on COVID-19–dedicated biomode units, emerged from this initiative. Once the number of patients with COVID-19 exceeded BCU capacity, BCU team members were deployed to the biomode units to address PPE safety and training, to augment existing bedside staffing models, and to provide operational and technical consultative services.

### Patient care and unit operations prior to COVID-19

The BCU was prepared to activate to care for patients with COVID-19 in part because of the development and implementation of a novel tool called the “BCU Readiness Scale and Checklist.”^12^ This tool was created in 2018 to identify barriers to successful unit activation and to allow the BCU team to proactively update unit protocols and infrastructure to mitigate those barriers. Readiness is measured not only in terms of functional clinical space (eg, a working negative pressure air-handling system) but also safe staffing ratios based on clinical acuity and adequate PPE supplies. Regular use of this checklist meant that the BCU was ready to activate for the first COVID-19 PUIs and confirmed cases.

In addition to preparing for a unit activation, the BCU team partnered with the Johns Hopkins Lifeline transport team to establish the JH Special Operations Transport Team (SORT). SORT consists of emergency medical technicians, paramedics, and critical care nurses who have undergone special training in infection control precautions. The SORT established protocols for both intra- and interhospital transport of patients with suspected or confirmed high-consequence pathogens and participated in full functional exercises and the transport of PUIs to the BCU. They have worked with the US military to provide ground transport operations during large federal drills. SORT has also become an operational and educational resource for other transportation services in Maryland and Region 3 looking to establish their own high-consequence pathogens transport protocols.

The BCU also worked closely with OEM from 2017 to 2019 to establish a unit-specific incident command structure. The incident command structure model was used in BCU fully functional exercises with great success and became a model for the health system in terms of the utilization of incident command principles in the response to preparedness emergencies including mass casualty events and infectious disease outbreaks.^13^

### Patient care and unit operations during COVID-19

From February 29 through March 20, 2020, the BCU team managed the first 3 persons under investigation (PUIs) and the first 11 confirmed cases of COVID-19 at JH Health System. The BCU team activated and decommissioned a total of 3 times, caring for 10 patients in the BCU’s physical space with the remaining 4 patients located on a sister medical unit in airborne infection isolation rooms staffed by BCU team members. Several of these patients required hemodialysis and/or critical care services necessitating the addition of new staff members, such as dialysis technicians, to the BCU team. All new staff underwent comprehensive just-in-time training by existing BCU staff members prior to caring for patients. Previous fully functional exercises demonstrated that this type of training was effective in limiting the likelihood of healthcare worker exposure to highly infectious diseases.^8^

For each activation of the BCU for COVID-19, the time interval between the decision for unit activation and unit readiness, defined as the physical and functional ability to safely admit patients to the BCU, was <90 minutes. As a regional treatment center, the BCU also met its 8-hour goal between unit activation notice and patient admission to JH Health System for each of the COVID-19 activations. Safe, organized, and expedited transport of patients for admission to the JH BCU from the JH Emergency Department and outside hospitals was provided by Lifeline SORT. Transportation strategies were updated using a multidisciplinary after-action “hot wash” after each admission. The BCU partnered with SORT to expand the safety officer role during patient admission transports, which was later expanded to all inter- and intrahospital COVID-19 patient transports. BCU team members also served as these transport safety officers (TSOs) as demand increased which provided time for Lifeline and JHH to implement a TSO education program to increasing overall transport capacity.

Upon activation, the implementation of an incident command structure was necessary to maintain situational awareness and to facilitate planning for the COVID-19 patient surge throughout the health system. The BCU command directly communicated relevant clinical information, safety issues, and frontline experiences to the hospital and health system incident command centers. Through this incident command structure, the hospital was able to stand up 14 biomode units to provide care for patients with COVID-19, and this model was later expanded to other health system hospitals to respond to the surge of COVID-19 patients throughout the JH Health System. The first 3 weeks of COVID-19 care lead by the BCU team provided the health system with the necessary time to create the physical infrastructure to have negative pressure on these biomode units and for the BCU and HEIC teams to properly train additional staff in the safe care of patients with COVID-19.

Once the number of patients exceeded the capacity of the BCU team at the end of March 2020, the BCU team merged with other units to continue providing frontline care. At this point, the BCU donated its 7-day par supply of viral hemorrhagic fever PPE to the health system response, including 1,000 complete PPE ensembles and 25 powered air purifying respirators (PAPRs). BCU and HEIC team members continued to serve as a vital resource for training and support of the biomode units’ infection prevention and clinical care protocols.

### Research and innovation prior to COVID-19

Since its creation in 2015, the BCU has sought to advance the field of containment care through innovative research projects that relate to medical countermeasures development, infection prevention, human factors engineering, environmental engineering, PPE, and bioethics. Shortly after opening the unit, the BCU team created and validated a process to ensure that autoclaves effectively decontaminate waste from patients infected with category A pathogens such as Ebola.^14^ In partnership with JHPIEGO (a non-profit affiliate of Johns Hopkins Health) and the JH Center for Bioengineering Innovation and Design (CBID), the BCU team helped to design and test a novel self-doffing PPE coverall.^15^ Of particular relevance to the current COVID-19 pandemic, the BCU team, in collaboration with the JH Applied Physics Lab, used a novel fluorescent microbead system to simulate the movement of infectious particles through a biocontainment environment and to assess the impact of environmental control systems and patient care protocols on the movement of these infectious simulants.^16,17^ In partnership with HEIC, the BCU team combined these infectious simulants with more traditional methods to enhance the detection of self-contamination during the doffing process, and to validate the use of a trained observer in the doffing process for viral hemorrhagic fevers.^18,19^ The BCU team partnered with the JH Berman Bioethics Institute to explore the ethical, legal, and social implications of genomic research on containment care.^20^ In 2016, the BCU team also partnered with the other regional treatment centers to form the Special Pathogens Research Network (SPRN) to improve the capacity for research to be conducted during an infectious disease outbreak.^21^

### Research and innovation during COVID-19

During the COVID-19 pandemic, the BCU continued to play an active role in research on highly infectious diseases. Using the central institutional review board established through the SPRN, the BCU team led the Johns Hopkins arm of the Adaptive COVID-19 Treatment Trial (ACTT-1), which showed that remdesivir compared to placebo reduced the time to recovery in hospitalized COVID-19 patients.^22^ The BCU team helped to establish the JH-CROWN registry which includes all COVID-19 patients seen in the JH Health System. This registry will be used to better understand the pathobiology of COVID-19 and to inform the clinical care of COVID-19 patients.^23^ In tandem with the JH CROWN registry, the BCU team efficiently implemented multiple protocols that allowed for the collection of residual clinical and prospectively collected research specimens. The BCU team also established a longitudinal COVID-19 cohort that aligns with the global ISARIC research protocol.^24^ This cohort will allow more in-depth exploration into the pathophysiology of COVID-19 by correlating patient phenotype with physiologic samples, such as blood, urine, and bronchoalveolar lavage fluid, in addition to serving all the downstream sample needs of the basic and translational researchers across Johns Hopkins.

In conclusion, the JH BCU played a critical role in the JH Health System response to COVID-19. Through its preparedness planning, frequent training activities, robust educational programs, research activities, and integration into a broader hospital and health system incident command structure, the JH BCU demonstrated the value of having a dedicated highly infectious disease unit and team during a pandemic surge. The creation of similar units and trained teams beyond the existing regional treatment centers could enhance health system preparedness and resilience for the continued fight against COVID-19 and for future infectious disease outbreaks.
